# Expression Profiles of hsa-miR-4715-3p and hsa-miR-5000-3p in Breast Cancer: Findings from Functional and Regulatory Network Analyses

**DOI:** 10.3390/diagnostics16193199

**Published:** 2026-10-02

**Authors:** Aysegul Cihangor, Deniz Mihcioglu, Turkan Gurer, Alper Aytekin, Filiz Ozbas Gerceker

**Affiliations:** 1 Department of Biology, Faculty of Art and Science, Gaziantep University, Gaziantep 27310, Turkey; aysegulcihangor@gmail.com (A.C.); filiz.gerceker@gmail.com (F.O.G.); 2 Department of Nutrition and Dietetics, Faculty of Health Science, SANKO University, Gaziantep 27090, Turkey; denizmihcioglu@hotmail.com; 3 Department of General Surgery, School of Medicine, Gaziantep University, Gaziantep 27310, Turkey; aytekinalper83@hotmail.com

**Keywords:** breast cancer, hsa-miR-4715-3p, hsa-miR-5000-3p, expression, bioinformatics analyses

## Abstract

**Background/Objective:** In this study, we aimed to determine the expression profiles of hsa-miR-4715-3p and hsa-miR-5000-3p in breast tumor tissues and to investigate their possible biological and regulatory relationships through integrated bioinformatics analyses. **Methods:** Tumor and adjacent non-tumor breast tissues were obtained from 38 patients with breast cancer, and the expression levels of hsa-miR-4715-3p and hsa-miR-5000-3p were determined using qRT-PCR. To evaluate the functional characteristics of miRNAs, GO and KEGG enrichment analyses were performed. Additionally, to conduct a more comprehensive evaluation of the regulatory networks associated with both miRNAs, TF–miRNA interactions, target genes, TF functions, and disease associations were examined. **Results:** hsa-miR-4715-3p was significantly downregulated, whereas hsa-miR-5000-3p was markedly upregulated in tumor tissues compared with adjacent non-tumor tissues (both *p* < 0.001). GO analysis revealed enrichment in metabolic, biosynthetic, and gene regulatory processes, while KEGG analysis highlighted the TGF-beta signaling pathway. TF network analysis identified shared regulatory TFs for both miRNAs, all of which were associated with breast cancer. **Conclusions:** hsa-miR-4715-3p and hsa-miR-5000-3p showed distinct expression patterns in breast tumor tissues. Their association with signaling and transcriptional networks relevant to breast cancer suggests that these miRNAs may represent underexplored components of breast cancer biology. The findings support further investigation of hsa-miR-4715-3p and hsa-miR-5000-3p; however, validation in independent patient cohorts and functional studies are necessary to establish their biological and potential clinical significance.

## 1. Introduction

Breast cancer is the most frequently diagnosed malignancy among women worldwide and continues to be the leading cause of cancer-related death among women worldwide. According to the most recent global cancer statistics, approximately 2.3 million women were diagnosed with breast cancer in 2022, and around 670,000 deaths were attributed to this disease [1]. Despite significant advancements in current screening programs, developed molecular diagnostic methods, and treatment strategies, the global burden of breast cancer continues to rise worldwide, and it is anticipated that both incidence and mortality rates will significantly increase in the coming years [2]. Breast cancer is a multifactorial disease in which genetic predisposition, hormonal status, reproductive history, aging, lifestyle, obesity, alcohol consumption, smoking, and environmental factors all play a role [3]. Due to its distinct biological heterogeneity and variable clinical course among patients, elucidating the molecular underpinnings of the disease and identifying reliable molecular biomarkers continue to be among the primary goals of breast cancer research [4,5]. Breast cancer is a heterogeneous group of diseases consisting of various molecular and histopathological subtypes that show significant differences in prognosis, response to treatment, and clinical behavior [6,7]. Tumor size, histological subtype, presence of lymph node involvement, hormone receptor status, and HER2 expression are indispensable traditional clinical and pathological data in diagnosis and treatment planning, but they often fall short in fully explaining tumor heterogeneity and accurately predicting the clinical course of the disease [8]. Therefore, especially in recent years, the identification of new molecular biomarkers that can facilitate early diagnosis, enable more accurate prognosis determination and monitoring, and contribute to personalized treatment strategies has become even more important, and research in this field has gained momentum [9].

The human genome comprises 3 × 10^9^ base pairs. Approximately 2000 microRNAs (miRNAs) are now annotated in the human genome; yet, each mature miRNA measures just 20–25 base pairs in length, constituting a minuscule portion of the genome’s overall sequence length. miRNAs can participate in the inhibition or stimulation of malignancies [10]. Although miRNAs constitute a very small portion of the human genome, they can regulate the expression of numerous protein-coding genes and play a role in many fundamental cellular processes, including cell proliferation, differentiation, apoptosis, angiogenesis, immune response, and the maintenance of genomic stability [11,12]. A single miRNA can have multiple target genes and can regulate a large number of target genes simultaneously. With these characteristics, miRNAs can significantly affect the complex cellular signaling networks that play an important role in cancer development [13]. Translational studies in the literature, which are increasing in number every day, show that irregularities in miRNA expression are one of the fundamental molecular characteristics of human cancers [14]. Depending on the target genes they regulate and the cellular environment they are in, miRNAs can exhibit oncogenic (oncomiR) or tumor-suppressive properties. These bidirectional regulatory properties once again highlight the critical importance of miRNAs in cancer biology [15]. To date, a large number of miRNAs have been identified that play a role in the development of breast cancer. Among these, hsa-miR-21, hsa-miR-155, hsa-miR-182, and hsa-miR-22 are the main oncomiRs that support tumor development by targeting genes associated with cell proliferation, apoptosis, epithelial–mesenchymal transition, angiogenesis, and immune regulation [16,17,18,19]. In contrast, miRNAs such as the let-7 family, hsa-miR-145, hsa-miR-146 and hsa-miR-335 exhibit tumor-suppressive functions by inhibiting oncogenic signaling pathways associated with the cell cycle, cell migration, and metastasis [20,21,22,23]. Despite the increasing number of miRNAs involved in the development and progression of breast cancer, the functions of some miRNAs in breast tumor biology have not yet been sufficiently elucidated. One of these miRNAs, hsa-miR-4715-3p, has limited associations with various cancer types; however, findings in the literature suggest that this miRNA may play a role in different molecular mechanisms related to carcinogenesis. When these studies are examined, hsa-miR-4715-3p’s involvement in upper gastrointestinal adenocarcinomas [24] and lung cancer [25] is observed. Although studies on the role of another RNA molecule, hsa-miR-5000-3p, in cancer biology are limited, current findings suggest that this miRNA may play a role in molecular processes associated with tumor progression, metastasis, and treatment resistance. Among these, a study conducted in 2021 suggested that hsa-miR-5000-3p is involved in the progression of drug resistance in colorectal cancer and that this miRNA could be a new biomarker for the progression of drug resistance in colorectal cancer [26]. As a result of preliminary research conducted using some bioinformatics analysis tools, we found that some of the target genes of hsa-miR-4715-3p and hsa-miR-5000-3p are involved in the biological pathways associated with the development of breast cancer. To the best of our knowledge, no previous study has investigated the expression profiles of hsa-miR-4715-3p and hsa-miR-5000-3p in breast cancer tissues. Additionally, there is quite limited information on the target genes and biological pathways associated with hsa-miR-4715-3p and hsa-miR-5000-3p, as well as on the transcriptional regulatory networks involving these miRNAs and their associated TFs. Therefore, the present study aims to investigate the functional roles of hsa-miR-4715-3p and hsa-miR-5000-3p in breast cancer.

## 2. Materials and Methods

### 2.1. Study Subjects

The study included 38 female patients aged 18 and over who were diagnosed with primary breast cancer confirmed by histopathological examination and underwent surgical treatment in the Department of General Surgery at Gaziantep University Faculty of Medicine, and tissue samples from the tumors and adjacent healthy tissues of these patients were collected. Patients who had not received chemotherapy, radiotherapy, or other systemic anticancer treatments before surgery were eligible for inclusion. Additional inclusion criteria were the availability of paired tumor and adjacent non-tumor breast tissues obtained during surgery, sufficient sample integrity and yield for molecular analyses, and complete clinicopathological data. Following an explanation of the study objectives and procedures, written informed consent was obtained from all patients prior to enrollment. Patients without a histopathological diagnosis of breast cancer were excluded. Additional exclusion criteria included a previous or concurrent malignancy other than breast cancer, autoimmune or inflammatory diseases, cardiovascular disease within the previous six months, active infection, insufficient clinicopathological data, and lack of written informed consent. Tissue samples from each individual were placed in tubes containing RNAlater^®^ solution (Catalog No. AM7021; Invitrogen, Thermo Fisher Scientific, Inc., Carlsbad, CA, USA) and stored at −80 °C. The Clinical Research Ethics Committee of Gaziantep University approved this study on 23 February 2022, under decision number 2021/402.

### 2.2. miRNA Isolation of Tissue Samples of Patients with Breast Cancer

RNA isolation was performed using the Hybrid-R™ miRNA Isolation Kit (Catalog No: 325-150; GeneAll Biotechnology Co., Ltd., Seoul, Republic of Korea) according to the manufacturer’s instructions. The concentration and purity of the isolated RNA samples were determined using a NanoDrop spectrophotometer (Maestrogen, Hsinchu, Taiwan). The concentration of each RNA sample was determined using the A260 absorbance value, and its purity was assessed using the A260/A280 and A260/A230 absorbance ratios. Samples with an A260/A280 ratio between 1.8 and 2.1 were considered high-purity RNA. The integrity of the RNA samples was confirmed by agarose gel electrophoresis at a 1% concentration. After isolation, RNA samples were stored at −80 °C until used in subsequent analyses.

### 2.3. cDNA Synthesis by the Reverse Transcriptase PCR

The synthesis of miRNA-specific cDNA from isolated RNA samples was performed using the TaqMan™ Advanced miRNA cDNA Synthesis Kit (Catalog No. A28007; Applied Biosystems, Thermo Fisher Scientific, Waltham, MA, USA) according to the protocol recommended by the manufacturer. In the synthesis of cDNAs, the steps included the addition of a poly(A) tail to mature miRNAs and the ligation of adapters, followed by the reverse transcription reaction. The cDNA samples were stored at −20 °C until the next application.

### 2.4. Quantitative Real-Time Polymerase Chain Reaction (qRT-PCR)

Quantitative Real-Time PCR (qRT-PCR) was performed to determine the expression levels of hsa-miR-4715-3p and hsa-miR-5000-3p, and this analysis was carried out using StepOne and StepOne-Plus Real-Time PCR Systems (Applied Biosystems, USA). TaqManTM Advanced miRNA Assay (Catalog No. A25576; Applied Biosystems, Thermo Fisher Scientific, Waltham, MA, USA), TaqManTM Primer Probe (Thermo Fisher Scientific, Waltham, MA, USA) and TaqManTM Fast Advanced Master Mix (Catalog No. 4444557; Applied Biosystems, Thermo Fisher Scientific, Waltham, MA, USA) were used for qRT-PCR. *RNU6* was used as the housekeeping gene. qRT-PCR was performed in technical triplicate for each sample, and the mean Ct values were utilized to determine gene expression levels. The data obtained from the qRT-PCR analysis were evaluated using the 2^−ΔΔCt^ method [27].

### 2.5. Functional Enrichment Analysis of hsa-miR-4715-3p and hsa-miR-5000-3p

In order to more comprehensively evaluate the biological significance of the experimentally determined miRNA expression changes, functional enrichment analyses were conducted for hsa-miR-4715-3p and hsa-miR-5000-3p. In this context, the potential targets of miRNAs and the biological processes associated with these targets were investigated using the DIANA-miRPath v3.0 platform. In determining miRNA-target gene relationships, DIANA-TarBase data, which includes experimentally validated interactions, and the microT-CDs prediction algorithm were utilized [28]. To evaluate the cellular functions and molecular pathways of the identified target genes, enrichment analyses based on Gene Ontology (GO) and Kyoto Encyclopedia of Genes and Genomes (KEGG) were conducted [29,30]. Within the scope of GO analysis, the target genes were evaluated under three main categories: biological process (Biological Process, BP), molecular function (Molecular Function, MF), and cellular component (Cellular Component, CC). The KEGG analysis was used to identify the cellular and molecular signaling pathways that hsa-miR-4715-3p and hsa-miR-5000-3p might be associated with through their target genes. Thus, the aim was to evaluate the experimental expression findings within a functional framework, focusing on the potential regulatory roles of these miRNAs in breast cancer biology. In enrichment analyses, *p* < 0.05 was considered the threshold for statistical significance.

### 2.6. Analysis of Regulatory Interactions Between hsa-miR-4715-3p, hsa-miR-5000-3p, and Transcription Factors

To evaluate the potential regulatory roles of hsa-miR-4715-3p and hsa-miR-5000-3p in breast cancer not only at the target gene level but also within a broader transcriptional regulation framework, transcription factors (TFs) associated with these miRNAs were investigated. In determining the miRNA–TF regulatory relationships, the TransmiR v3.0 database, which compiles interactions based on experimental evidence, was utilized [31]. As a result of this analysis, the TRRUST v2.0 database was used to examine the genes and regulatory connections that the identified TFs could influence downstream [32]. Additionally, this database was utilized to extract TF-related disease information and to assess the correlations between the identified TFs and specific disease phenotypes. To further evaluate the biological significance of the TFs identified in the analyses as targets of both miRNAs, data from The Human Protein Atlas were utilized [33]. The identified TFs were examined in terms of their molecular functions, associated biological processes, and cancer-related pathways. Through this holistic approach, the aim was to elucidate the potential miRNA-TF-target gene regulatory networks involving hsa-miR-4715-3p and hsa-miR-5000-3p in breast cancer.

### 2.7. Statistical Analysis

Statistical evaluations were conducted using the SPSS 22.0 (SPSS Inc., Chicago, IL, USA) program. Continuous variables were summarized as mean ± standard deviation (SD). The relative expression levels of hsa-miR-4715-3p and hsa-miR-5000-3p were calculated using the 2^−ΔΔCt^ method. In the evaluation of expression differences between tumor tissues and matched non-tumor tissues from the same patients, ΔCt values were used as the basis. The distribution characteristics of numerical data were evaluated using the Kolmogorov–Smirnov and Shapiro–Wilk tests. Since the ΔCt values showed a normal distribution, the differences between paired tumor and non-tumor tissues were analyzed using a paired sample t-test. In all analyses, *p* < 0.05 was considered statistically significant.

## 3. Results

### 3.1. Clinicopathological Features of Breast Cancer Patients

All participants in the study cohort were women. The vast majority of the patients (71.05%) were 55 years old or younger, while 28.95% were over 55 years old. Only 7.90% of the patients had a family history of breast cancer. The tumor was evenly distributed, with 50% on the right side and 50% on the left side. More than half of the patients (52.64%) had histological grade stage 3 disease. 52.63% of the patients did not show metastatic lymph nodes, 31.57% presented with several metastatic lymph nodes, and 15.78% presented with one metastatic lymph node. The majority of the patients (92.10%) did not show distant metastasis. Regarding molecular phenotype, 12 (31.58%) patients were classified as Luminal A, 14 (36.84%) as Luminal B, 10 (26.32%) as HER2-positive, and 2 (5.26%) as triple-negative breast cancer. The demographic, clinicopathological features and molecular phenotypes of patients with breast cancer included in the study are summarized in Table 1.

### 3.2. Expression Analyses of hsa-miR-4715-3p and hsa-miR-5000-3p in Breast Cancer Tissues

**Figure 1:**
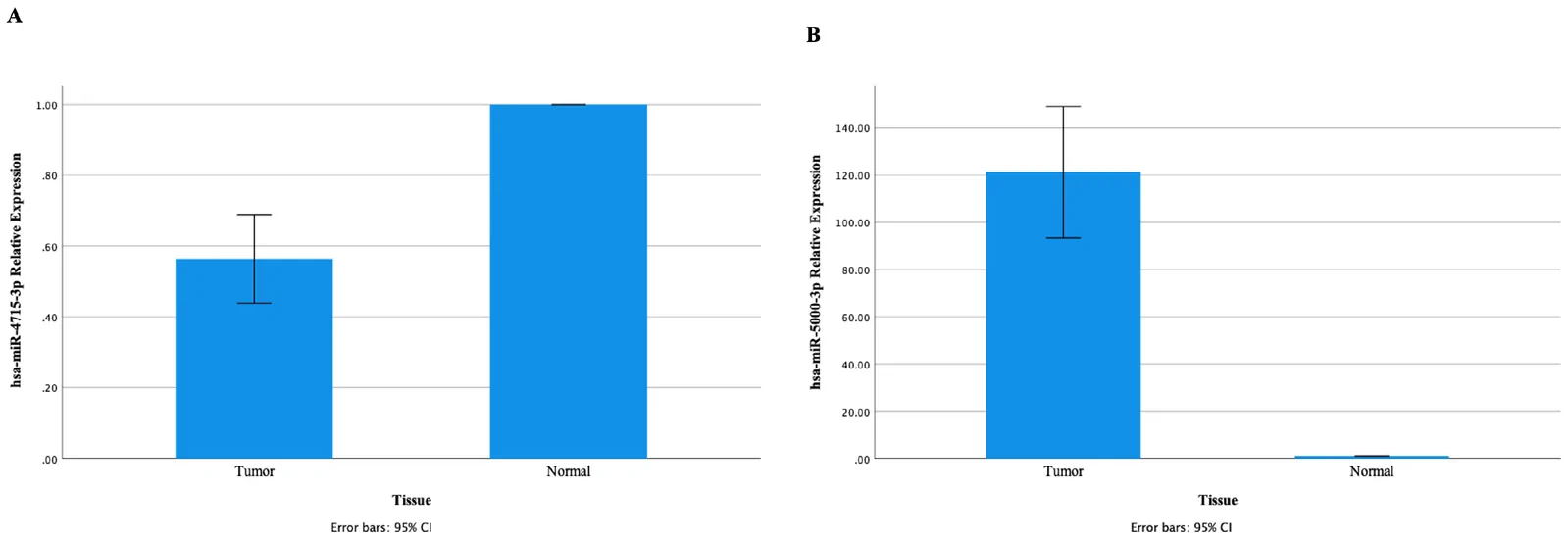
Differential expression of hsa-miR-4715-3p and hsa-miR-5000-3p in breast tumor and matched non-tumor tissues. (**A**) hsa-miR-4715-3p expression levels. (**B**) hsa-miR-5000-3p expression levels. *p* < 0.001.

The expression levels of hsa-miR-4715-3p and hsa-miR-5000-3p were evaluated in matched tumor and non-tumor breast tissues. It was determined that the expression of hsa-miR-4715-3p was significantly lower in tumor tissues compared to non-tumor tissues (*p* < 0.001), and the fold-change value was calculated as 0.56 ± 0.39 (Figure 1A). In contrast, hsa-miR-5000-3p expression was significantly upregulated in tumor tissues compared to non-tumor tissues (*p* < 0.001). The fold change value for hsa-miR-5000-3p was determined to be 121.33 ± 86.01 (Figure 1B). Overall, these two miRNAs exhibited opposite expression profiles in breast cancer tissues; hsa-miR-4715-3p was downregulated, while hsa-miR-5000-3p was significantly upregulated.

### 3.3. GO and KEGG Enrichment Analysis of hsa-miR-4715-3p and hsa-miR-5000-3p

In the bioinformatics analysis stage of the study, Gene Ontology (GO) enrichment analysis was conducted using the DIANA-miRPath database to investigate the potential biological functions of target genes associated with hsa-miR-4715-3p and hsa-miR-5000-3p. The significantly enriched GO terms were categorized under three main headings. These categories consisted of Biological Process (BP), Cellular Component (CC), and Molecular Function (MF). In the BP category, it was determined that the target genes are primarily associated with cellular nitrogen compound metabolic processes, biosynthetic processes, gene expression, small molecule metabolic processes, and cellular protein modification processes. In the CC category, organelle emerged as a term showing significant enrichment. In the MF analysis, ion binding, enzyme regulatory activity, nucleic acid binding transcription factor activity, and enzyme binding were among the significantly enriched molecular functions (Table S1).

To identify the signaling pathways associated with the target genes of hsa-miR-4715-3p and hsa-miR-5000-3p, a KEGG pathway enrichment analysis was conducted. Consequently, significant enrichment was observed in various pathways, including thyroid hormone synthesis (hsa04918), TGF-beta signaling pathway (hsa04350), cocaine addiction (hsa05030), lysine degradation (hsa00310), and arrhythmogenic right ventricular cardiomyopathy (ARVC) (hsa05412). Among these pathways, the highest statistical significance was observed in the thyroid hormone synthesis pathway (*p* = 1.12 × 10^−5^), followed by the TGF-beta signaling pathway (*p* = 4.09 × 10^−4^). The significantly enriched pathways and their associated target genes are presented in Table 2.

**Figure 2:**
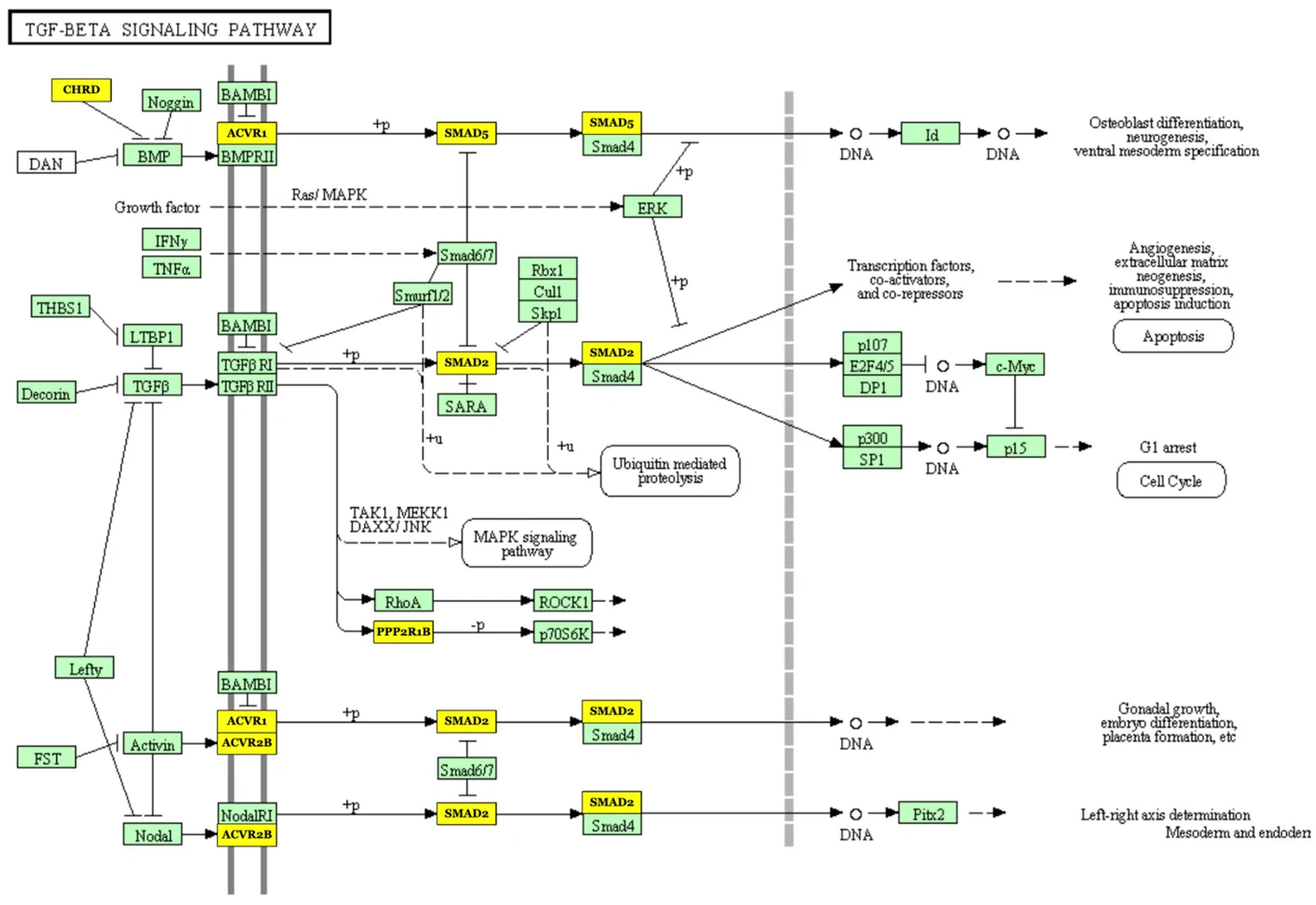
Mapping of target genes associated with hsa-miR-4715-3p (*SMAD2*) and hsa-miR-5000-3p (*CHRD*, *ACVR1*, *SMAD5*, *PPP2R1B*, and *ACVR2B*) within the TGF-β signaling pathway.

Due to its importance in cellular processes associated with breast cancer, the TGF-beta signaling pathway was also examined. In this pathway, six target genes associated with miRNAs were identified: SMAD2, CHRD, ACVR1, ACVR2B, SMAD5, and PPP2R1B. Among these, CHRD, ACVR1, ACVR2B, SMAD5, and PPP2R1B were associated with hsa-miR-5000-3p, while SMAD2 was associated with hsa-miR-4715-3p. The positions of these target genes in the TGF-beta signaling pathway and their relationships with the two miRNAs are shown in Figure 2.

**Figure 3:**
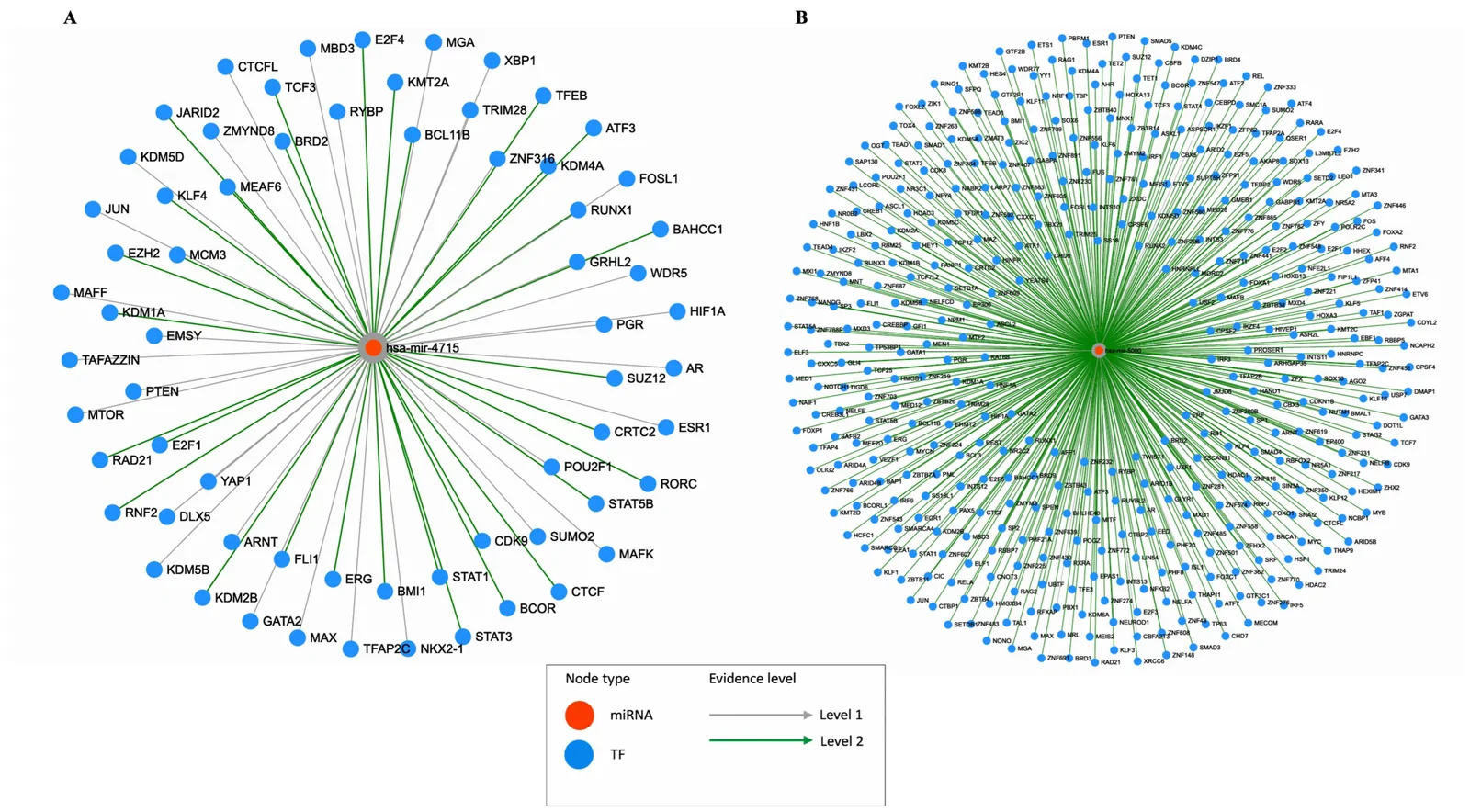
Transcription factor (TF) regulatory profiles associated with hsa-miR-4715-3p and hsa-miR-5000-3p. (**A**) TFs associated with hsa-miR-4715-3p; (**B**) TFs associated with hsa-miR-5000-3p.

To evaluate the transcriptional regulatory structure associated with hsa-miR-4715-3p and hsa-miR-5000-3p in more detail, TF–miRNA regulatory interactions were analyzed using the TransmiR v3 database. First, the TF profiles associated with each miRNA were examined separately and shown in Figure 3. Comparison of the regulatory profiles identified a set of shared regulatory TFs associated with both hsa-miR-4715-3p and hsa-miR-5000-3p, including AR, ARNT, ATF3, CTCF, E2F1, E2F4, ELF4, ERG, ESR1, EZH2, FLI1, GATA2, HIF1A, JUN, KLF4, MAX, PGR, POU2F1, RUNX1, STAT1, STAT3, STAT5B, TCF3, and TFAP2C. Subsequently, the downstream target genes and disease associations of these shared regulatory TFs were analyzed using TRRUST v2.0, while their functional characteristics were assessed utilizing data from The Human Protein Atlas. Overall, it was observed that these TFs play roles in a wide range of biological processes, including cell proliferation and differentiation, regulation of the cell cycle, apoptosis, transcriptional and epigenetic regulation, response to hypoxia and cellular stress, angiogenesis, hormone-dependent signaling mechanisms, and immune and inflammatory responses. Among the shared regulatory TFs, some were particularly noteworthy in terms of regulatory processes associated with breast cancer. While ESR1 and PGR were associated with estrogen- and progesterone-dependent transcriptional regulation, respectively, HIF1A was linked to hypoxia adaptation and angiogenesis. Additionally, STAT3 plays a role in the regulation of cell proliferation and survival through cytokine- and growth factor-dependent signaling, while TFAP2C is associated with chromatin-related transcriptional regulation and the epigenetic regulation of ESR1 in breast cancer. Disease association analysis also showed that a significant portion of the shared regulatory TFs are associated with malignant neoplasms and that breast cancer is notably repeated among the associated diseases. The downstream target genes, functional characteristics, and disease associations of the regulatory TFs shared by hsa-miR-4715-3p and hsa-miR-5000-3p are summarized in Table S2.

## 4. Discussion

Malignancy is acknowledged as the result of abnormal and/or hereditary genetic alterations in germline or somatic cells. Breast cancer is similarly induced by genetic and environmental causes, leading to the accumulation of mutations in particular genes [34]. Breast cancer is the major cancer type among women and the primary cause of mortality in women aged 40–55. It continues to be a significant global health issue [35]. The disease exhibits a wide range of complicated and diverse subgroups, each with distinct genetic features and clinical pathways. Breast tumors are classified into several molecular subgroups based on the expression levels of Human epidermal growth factor receptor 2 (HER2), progesterone, and estrogen. Alongside genomic abnormalities in breast cancer, epigenetic alterations can interfere with normal cellular functions in breast cancer and other neoplasms [36]. Increasing the efficacy of treatment for women with breast cancer requires early detection of malignant lesions and evaluation of their prognosis [35]. The most significant molecular markers associated with breast cancer are estrogen receptor, progesterone receptor, HER2, and the Mib1/Ki-67 proliferation index. These markers are beneficial for assessing therapy efficacy and for the early identification of recurrence or metastasis. Moreover, miRNAs contribute intricately to the formation and proliferation of breast gland tissue [37]. Multiple studies have demonstrated that miRNAs exhibit aberrant expression in tissue and blood samples from women diagnosed with breast cancer [38,39,40]. miRNA dysregulation exhibits significant variation across distinct temporal stages in tissues, such as normal breast tissue, precancerous tissue, and malignant tissue. miRNAs may serve as possible indicators for distinguishing between these stages [35]. The capacity to quantify miRNA expression in diverse biological specimens (tissue, blood) enhances their predictive value [41,42].

The present study identified contrasting expression patterns of hsa-miR-4715-3p and hsa-miR-5000-3p in breast cancer tissues, characterized by decreased hsa-miR-4715-3p and increased hsa-miR-5000-3p expression. In concordance with our study, hsa-miR-4715-3p expression has previously been shown to be significantly downregulated in upper gastrointestinal system malignancies. Interestingly, this downregulation has been linked to an epigenetic mechanism, as increased CpG methylation in the upstream region of hsa-miR-4715-3p was observed in upper gastrointestinal tumors, whereas pharmacological inhibition of DNA methylation increased its expression [24]. This finding raises the possibility that epigenetic regulation may contribute to the reduced hsa-miR-4715-3p expression observed in our breast cancer tissues. However, as DNA methylation was not examined in the present study, whether a similar mechanism operates in breast cancer remains to be determined. In contrast, Yu and colleagues reported an increase in hsa-miR-4715-3p expression in lung cancer, associating this with elevated XPG mRNA levels [25]. Furthermore, the expression of hsa-miR-5000-3p was found to be significantly increased in breast tumor tissues compared to normal tissues. Xia et al. reported increased expression of several miRNAs, including hsa-miR-5000-3p, in HCT-15 and HT-29 colon cancer cell lines [43]. Chen et al. provided evidence for an upstream regulatory mechanism involving hsa-miR-5000-3p in laryngeal carcinoma. In their study, MIR22HG directly interacted with hsa-miR-5000-3p and negatively regulated its expression, while the transcriptional repressor E2F6 suppressed MIR22HG transcription [44]. This E2F6–MIR22HG regulatory relationship provides a possible mechanism through which hsa-miR-5000-3p expression may be altered in cancer cells. Although this mechanism has not been established in breast cancer, it raises the possibility that changes in upstream transcriptional and lncRNA-mediated regulation may contribute to the increased hsa-miR-5000-3p expression observed in our study. Zhuang et al., in another study, demonstrated that hsa-miR-5000-3p plays a supportive role in tumor development in colorectal cancer. In various colorectal cancer cell lines, the upregulation of hsa-miR-5000-3p has been reported to promote tumor proliferation and contribute to the development of resistance [26]. Taken together, these findings support the potential involvement of hsa-miR-5000-3p in tumor-associated processes, although the mechanisms underlying its increased expression and its functional role in breast cancer remain to be established.

Additional insights into the potential biological processes associated with the target genes of hsa-miR-4715-3p and hsa-miR-5000-3p were obtained through GO enrichment analysis. The GO enrichment profile was predominantly associated with metabolic, biosynthetic, and gene regulatory processes. In breast cancer, it is known that the reorganization of nucleotide, amino acid, and lipid synthesis, along with changes in energy utilization, contributes to tumor growth and progression [45]. Therefore, the enrichment of GO terms related to metabolism and biosynthesis in the analyses suggests that the regulatory networks associated with hsa-miR-4715-3p and hsa-miR-5000-3p may intersect with cellular programs that support breast tumor development. Among the other noteworthy findings are the enrichment of gene expression and cellular protein modification processes. Both transcriptional and post-transcriptional regulations are important components of breast cancer biology, and miRNAs contribute to this complex regulatory structure by targeting numerous genes and regulating their expression [46]. However, changes in protein modifications can affect the stability, intracellular localization, interactions, and activity of proteins, thereby altering the signaling and transcription programs involved in breast cancer [47]. Therefore, the enrichment observed in these terms suggests the idea that both miRNAs may be associated with multilayered regulatory mechanisms rather than a single biological process. The findings obtained from the Molecular Function (MF) analysis also support this assessment. The significant enrichment of nucleic acid binding transcription factor activity, along with enzyme binding and enzyme regulatory activity, suggests a close connection between the mechanisms that control transcription and protein activity and the predicted targets associated with miRNAs. It is well established that interactions between miRNAs and transcription factors can form regulatory networks that jointly control the expression of many different genes [48]. This finding is particularly significant in light of the identification of a shared regulatory TF group associated with both hsa-miR-4715-3p and hsa-miR-5000-3p in the regulatory network analysis conducted in the present investigation. Overall, the GO results reveal a regulatory structure encompassing various biological functions for hsa-miR-4715-3p and hsa-miR-5000-3p, providing a biological framework for evaluating the pathway relationships identified in the KEGG analysis.

Among the pathways identified by KEGG enrichment analysis, the TGF-β signaling pathway is particularly relevant in the context of breast cancer biology [49]. The TGF-beta signal plays a bidirectional role during tumor development, depending on cellular context. It can exhibit a tumor-suppressive effect by limiting cell proliferation and supporting cytostatic/apoptotic responses in normal and premalignant epithelial cells. However, with tumor progression, cancer cells can become less sensitive to these growth-suppressive effects and can utilize TGF-beta signaling to support epithelial–mesenchymal transition (EMT), invasion, metastatic spread, and the remodeling of the tumor microenvironment [50]. The effects of TGF-beta on the tumor microenvironment can also contribute to immune evasion, creating an additional mechanism that supports cancer progression. When considered together, the enrichment observed in TGF-beta associated genes suggests a potential link between the regulatory networks associated with hsa-miR-4715-3p (target gene- SMAD2) and hsa-miR-5000-3p (target genes- CHRD, ACVR1, ACVR2B, SMAD5, and PPP2R1B) and the signaling mechanisms involved in breast cancer progression. However, these bioinformatics relationships do not show that hsa-miR-4715-3p or hsa-miR-5000-3p directly activate or suppress the TGF-β pathway. Instead, our findings highlight the TGF-β pathway as one of the candidate signaling networks that could mediate some of the biological effects of altered miRNA expression. To clarify this possibility, functional studies examining the effects of altering hsa-miR-4715-3p or hsa-miR-5000-3p levels on TGF-beta signaling are needed.

TFs and miRNAs can interact within interconnected regulatory networks, together determining the transcriptional and post-transcriptional control of cancer-related genes. Literature indicates that such transcription factor (TF)–miRNA regulatory relationships are also present in breast cancer, potentially contributing to the molecular heterogeneity of the disease [51,52]. One of the notable findings of our analysis was the identification of a group of shared regulatory TFs associated with both hsa-miR-4715-3p and hsa-miR-5000-3p. Furthermore, the observation that all these shared regulatory TFs are associated with breast cancer in the disease association analysis supports the potential biological significance of the identified regulatory network in this type of cancer (Table S2). Among these, ESR1, HIF1A, and STAT3 are particularly prominent due to their relationships with fundamental processes of breast cancer biology, such as hormone-dependent transcriptional regulation, adaptation to hypoxia, cell survival, and tumor progression. From the perspective of ESR1, the interaction between estrogen receptor signaling and miRNA-mediated regulation has been experimentally demonstrated in ER-positive breast cancer, supporting this finding [53,54]. Taken together, these findings suggest that hsa-miR-4715-3p and hsa-miR-5000-3p, despite their opposing expression profiles, may be associated with partially overlapping transcriptional regulatory networks in breast cancer. However, the functional significance and direction of these regulatory relationships require experimental validation.

Several limitations should be considered when interpreting our findings. First, this was a small-cohort, single-center study, which limits the generalizability of the results to broader breast cancer populations. Although the clinicopathological characteristics and molecular phenotypes of the patients were documented, subgroup analyses according to tumor stage, histological grade, lymph node status, and molecular phenotype were not performed because of the small cohort size and the resulting small and uneven patient groups, particularly the limited number of triple-negative cases. Therefore, whether the magnitude of hsa-miR-4715-3p and hsa-miR-5000-3p expression changes, target-gene associations, and transcriptional regulatory relationships differ according to clinicopathological characteristics and molecular phenotypes remains to be investigated in larger and more balanced cohorts. Another limitation concerns the bioinformatic component of the study. Although integrating the expression data with GO, KEGG, and TF network analyses provided a broader biological context for these miRNAs, the identified regulatory relationships remain computationally supported associations rather than experimentally validated functional interactions. Studies conducted on larger and independent patient groups, supported by the validation of selected targets and in vitro functional experiments, will significantly contribute to a clearer understanding of the biological and potential clinical implications of the regulatory relationships that emerge here in breast cancer.

## 5. Conclusions

This study identified distinct expression patterns of hsa-miR-4715-3p and hsa-miR-5000-3p in breast cancer tissues. Bioinformatic analyses further indicated that both miRNAs are associated with regulatory networks involving diverse biological processes, signaling pathways, and transcriptional mechanisms. Among these findings, enrichment of the TGF-β signaling pathway, together with the identification of related genes including members of the SMAD family, suggests a potential link to signaling mechanisms relevant to breast cancer. In addition, hsa-miR-4715-3p and hsa-miR-5000-3p were associated with a group of shared regulatory TFs, all of which were linked to breast cancer in the disease-association analysis. These findings suggest that the two miRNAs, despite their distinct expression patterns, may converge on shared regulatory components relevant to breast tumor biology. Taken together, our experimental and computational findings highlight hsa-miR-4715-3p and hsa-miR-5000-3p as molecular candidates worthy of further investigation in breast cancer. Validation in independent patient cohorts and functional characterization of the proposed regulatory relationships will be necessary to determine their biological significance and potential value as biomarkers.

## Figures and Tables

**Table 1:** Demographic and clinicopathological characteristics of the breast cancer cohort.

Characteristics	Breast Cancer Patients *n* (%)	
Age (years)	≥55	11 (28.95)
	≤55	27 (71.05)
Family history of breast cancer	Yes	3 (7.90)
	No	35 (92.10)
Cigarette smoking	Yes	2 (5.27)
	No	36 (94.73)
Alcohol drinking	Yes	-
	No	38 (100)
Tumor location	Right sided	19 (50)
	Left sided	19 (50)
Histological grade	1	4 (10.53)
	2	14 (36.84)
	3	20 (52.63)
Metastatic Lymph Node	No	20 (52.63)
	1	6 (15.79)
	>1	12 (31.58)
Distant Metastasis	Yes	3 (7.90)
	No	35 (92.10)
Molecular phenotype	Luminal A	12 (31.58)
	Luminal B	14 (36.84)
	HER2-positive	10 (26.32)
	Triple-negative	2 (5.26)

**Table 2:** KEGG pathway enrichment profile of target genes associated with hsa-miR-4715-3p and hsa-miR-5000-3p.

Signaling Pathway	*p* Value	Target Genes	miRNAs
Thyroid hormone synthesis (hsa04918)	1.12 × 10^−5^	*CREB5, ATP1A2, PLCB1, TG, ADCY45*	hsa-mir-4715-3p and hsa-miR-5000-3p
TGF-beta signaling pathway (hsa04350)	0.0004	*SMAD2, CHRD, ACVR1, ACVR2B, SMAD5, PPP2R1B*	hsa-mir-4715-3p and hsa-miR-5000-3p
Cocaine addiction (hsa05030)	0.002	*CREB5, GRM3, JUN, MAOA, GNAI1, GRIN2B*	hsa-mir-4715-3p and hsa-miR-5000-3p
Lysine degradation (hsa00310)	0.017	*WHSC1L1, AASS, KMT2E, KMT2C*	hsa-mir-4715-3p and hsa-miR-5000-3p
Arrhythmogenic right ventricular cardiomyopathy (ARVC) (hsa05412)	0.017	*CDH2, CACNA2D3, DSC2, ITGA8, ITGA11, ITGA6, ITGB3*	hsa-mir-4715-3p and hsa-miR-5000-3p
Biosynthesis of unsaturated fatty acids (hsa01040)	0.007	*PECR*	hsa-miR-4715-3p
Glycosaminoglycan biosynthesis-heparan sulfate/heparin (hsa00534)	0.01	*EXT1, GLCE*	hsa-miR-4715-3p
D-Arginine and D-ornithine metabolism (hsa00472)	0.027	*DAO*	hsa-miR-4715-3p

## Data Availability

The datasets used and/or analyzed during the current study are available from the corresponding author on reasonable request.

## Supplementary Materials

The following supporting information can be downloaded at: https://www.mdpi.com/article/10.3390/diagnostics16193199/s1, Table S1: Functional classification of hsa-miR-4715-3p and hsa-miR-5000-3p target genes based on Gene Ontology (GO) enrichment analysis. Table S2: Downstream target genes, functional characteristics, and disease associations of shared regulatory transcription factors associated with hsa-miR-4715-3p and hsa-miR-5000-3p.

## Abbreviations

The following abbreviations are used in this manuscript:

qRT-PCR	Quantitative real time PCR
KEGG	Kyoto Encyclopedia of Genes and Genomes
Ct	Threshold cycle
miRNA	MicroRNA
GO	Gene Ontology
TF	Transcription Factor
HER2	Human epidermal growth factor receptor 2
EMT	Epithelial–mesenchymal transition
TGF-beta	Transforming growth factor beta
ESR1	Estrogen receptor 1
PGR	Progesterone receptor
STAT3	Signal transducer and activator of transcription 3
HIF1A	Hypoxia inducible factor 1 subunit alpha
JUN	Jun proto-oncogene, AP-1 transcription factor subunit
KLF4	KLF transcription factor 4
