# Supplementary material for: Expression Profiles of hsa-miR-4715-3p and hsa-miR-5000-3p in Breast Cancer: Findings from Functional and Regulatory Network Analyses
Source: Diagnostics (Basel). 2026 Oct 2;16(19):3199. doi: 10.3390/diagnostics16193199 (PMC13664427; doi:10.3390/diagnostics16193199)
Supplement: Supplementary file 1 [file diagnostics-16-03199-s001.zip › diagnostics-4532113-supplementary.pdf]

Table S1. Functional classification of hsa-miR-4715-3p and hsa-miR-5000-3p target genes based on Gene Ontology (GO) enrichment analysis

**Biological Process**		
**GO category**	**p value**	**Target genes**
Cellular nitrogen compound metabolic process (GO:0034641)	1.00 x 10^{-10}	*ZKSCAN5, GSTZ1, CCNT2, ZNF423, DIRAS1, PFAS , ZNF738, DKFZP686D09174, TOP3A, PHF8, USP49, ZNF616, DCTD, WASL, ATXN7L3, PAX6, LTA4H, VAX1, FUS, IRAK1BP1, RAD23B, SMAD2, ABCB8, TKT, HNRNPA1, ZNF76, WHSC1L1, ATP5F1, ZNF468, ZNF83, DDX23, ARF3, PTRF, UTP14C, AGO3, RORB, MPP1, RPSA, CASK, ZNF688, CREB5, ATP1A2, ZNF131, CTBP1, ABCG4, PRPF40A, AASS, PIWIL4, ADAT1, RAB6A, KAT6B, PSME3, ZNF117, HINFP, RPL37, ZNF562, SRCAP, TRPS1, DAO, AGAP1, HELQ, MLLT3, SPCS2, TARDBP, PGM3, COPS2, HOXB3, EZH2, MAP3K9, DDX3X, HACE1, POLR3E, ZNF107, NAA15, POU5F1B, ZNF746, HDAC9, SAFB, CHEK1, MYCBP2, ATP5S, KIF21B, SREBF2, DPF2, CDK8, FLII, DKC1, ZBTB37, SALL3, PIAS3, ZNF676, SP4, NFAT5, THRAP3, AGO1, HOMEZ, DCP2, N4BP2, ASTE1, PPARA, MAOA, ATRIP, ZNF493, MGMT, SNAPC5, HOXA5, FAN1, ZNF525, GUCY2C, CHD3, ZIC3, CSRNP3, SMAD5, ZNF138, AEN, SREK1, SATB1, KDM5A, PRPF38B, ZNF708, UBE2N, ZNF592, ZNF599, PCCB, ZNF157, TK2, FSHB, CDC7, KLHL31, LSM11, DDX55, VGLL2, ZNF646, ARID4B, NFE2L1, PLAG1, REV3L, PRKAG2, GUCY1A2, ABCA5, ZNF440, GRHPR, DTX1, KIF18B, PDE5A, BZW1, PRMT5, CHD5, TPP1, CTCF, RAB22A, ZNF709, ZNF619, PCYT1B, ZFHX4, GFPT2, TGIF2, ZNF483, BCL11B, OPA1, ABCC5, ZNF202, UBE2B, KMT2E, UGT1A8, ZNF674, AFF2, KMT2C, TBPL1, ADCY4, HMGB1, ZNF766, ABCC6, ZBTB25, NR2F2, GNAI1, TMF1, ZFAT*
Biosynthetic process (GO:0009058)	2.04 x 10^{-8}	*ZKSCAN5, GSTZ1, CCNT2, ZNF423, UGCG, PFAS, ZNF738, EXT1, PHF8, ZNF616, DCTD, WASL, SSR1, ST3GAL3, ATXN7L3, PAX6, LTA4H, VAX1, IRAK1BP1, SMAD2, TKT, SDC3, ZNF76, WHSC1L1, ATP5F1, ZNF468, ZNF83, ARF3, PTRF, RORB, RPSA, ZNF688, CREB5, ATP1A2, ZNF131, CTBP1, PECR, SGPL1, CYP46A1, KAT6B, MTMR4, ZNF117, RPL37, ZNF562, SRCAP, TRPS1, DAO, ETNK2, CLN8, MLLT3, SPCS2, TARDBP, PGM3, COPS2, EIF1AX, HOXB3, EZH2, MAP3K9, DDX3X, HACE1, POLR3E, NF107, NAA15, POU5F1B, ZNF746, HDAC9, SAFB, CHEK1, MYCBP2, ATP5S, HSPG2, SREBF2, DPF2 , CDK8, FLII, DKC1, ZBTB37, SALL3, PIAS3, ZNF676, SP4, NFAT5, THRAP3, AGO1, HOMEZ, HAS3, PPARA, QARS, MAOA, CIAPIN1, ATRIP, ZNF493, SNAPC5, HOXA5, ZNF525, GUCY2C, CHD3, ZIC3, CSRNP3, SMAD5, ZNF138, SATB1, KDM5A, ZNF708, ZNF592, ZNF599, PCCB, GLCE, ZNF157, TK2, FSHB, CDC7, KLHL31, HUNK, LSM11, VGLL2, ZNF646, ARID4B, NFE2L1, GPCPD1, PLAG1, REV3L, PRKAG2, GUCY1A2, ZNF440, ST8SIA2, DTX1, BZW1, PRMT5, TG, CHD5, CTCF, ZNF709, ZNF619, PCYT1B, ZFHX4, GFPT2, TGIF2, ZNF483, BCL11B, ABCC5, ZNF202, KMT2E, ZNF674, CPEB3, KMT2C, TBPL1, ADCY4, ZNF766, ZBTB25, MRPL32, NR2F2, TMF1, ZFAT*
Gene expression (GO:0010467)	0.0001	*CCNT2, DKFZP686D09174, SSR1, FUS, SMAD2, HNRNPA1, IGF2BP2, DDX23, PTRF, AGO3, RORB, RPSA, PSME3, RPL37, SPCS2, EIF1AX, POLR3E, CDK8, NXF1, AGO1, DCP2, PPARA, QARS, SNAPC5, LSM11, PRMT5, TGIF2, ZBTB25, TNRC6C*
Small molecule metabolic process (GO:0044281)	0.007	*GSTZ1, UGCG, DIRAS1, DKFZP686D09174, PFAS, EXT1, DCTD, WASL, ST3GAL3, LTA4H, ABCB8, TKT, SDC3, ATP5F1, DDX23, CALM1, ARF3, MPP1, CASK, ATP1A2, CTBP1, PECR, ABCG4, SGPL1, CYP46A1, AASS, RAB6A, MTMR4, PSME3, CA12, DAO, ETNK2, AGAP1, NDUFA5, CLN8,*

		AASDH, PGM3, DDX3X, ATP5S, KIF21B, HSPG2, SREBF2, PLCB1, HAS3, PPARA, GPD2, QARS, MAOA, CIAPIN1, ACOT11, CBR1, GUCY2C, CHD3, PRKAR2A, PCCB, GLCE, TK2, FSHB, MTHFR, LYPLA2, DDX55, GPCPD1, PRKAG2, GUCY1A2, ABCA5, GRHPR, KIF18B, PDE5A, TG, CHD5, PCYOX1L, RAB22A, PCYT1B, GFPT2, OPA1, ABCC5, UGT1A8, ADCY4, ABCC6, SLC46A1, TCN2, GNAI1, PFKFB3
Cellular protein modification process (GO:0006464)	0.02	*KSR2, PTPN3, GALNT7, DKFZP686D09174, ZNF738, EXT1, PHF8, USP49, UBR1, ST3GAL3, ATXN7L3, PAX6, SMAD2, WHSC1L1, MOK, CASK, CTBP1,RAB6A, KAT6B, MTMR4, PSME3, SRCAP, FES, PTPN21, PTPN1, PPP6C, TNKS2, PGM3, COPS2, RET, EZH2, MAP3K9, HACE1, NAA15, FKBP9, ACVR1, ABI2, HDAC9, CHEK1, MYCBP2, STK39, CDK8, ACVR2B, NAA50, PIAS3, PIGP, GUCY2C, ACVRL1, TRIB2, UBR2, UBE2N, CDC7, HUNK, DUSP3, RNF146, USP35, TSPAN17, CBLL1, USP14, TNIK, ST8SIA2, DTX1, CDK20, PRMT5, CHD5, MKRN3, ZRANB1, GFPT2, OTULIN, RPRD1A, PRKG1, UBE2B, KMT2E, SGK3, KMT2C, TRIM32, C1GALT1C1, GLYCTK, DUSP19, ILK*

Cellular Component

**GO category**	**p value**	**Target genes**
Organelle (GO:0043226)	4.17×10^{-17}	*ZKSCAN5, GSTZ1, CCNT2, ESPL1, HSPA13, CDIP1, UBXN7, CDKN2C, PTPN3, ZNF423, NAGA, GALNT7, CDH2, YIPF3, DKFZP686D09174, PFAS, DSC2, ZNF738, FXYD3, EXT1, TOP3A, PHF8, CLSTN3, USP49, ZNF616, RRAGD, DCTD, CYFIP2, WASL , SSR1, ATXN7L3, PAX6, LTA4H, FUS, IRAK1BP1, TM9SF1, RAD23B, SMAD2, KPRP, ABCB8, TKT, STAM, HNRNPA1, ZNF76, DND1, IGF2BP2, WHSC1L1, ADD2, SKIDA1, ATP5F1, ZNF468, ZNF83, DDX23, CALM1, ARF3, HAP1, PTRF, ITGA8, UTP14C, RORB, MORC4, RPSA, CASK, ZNF688, SPATA2, CREB5, ATP1A2, ZNF131, CTBP1, PECR, PRPF40A, PVR, SGPL1, CYP46A1, AASS, PIWIL4, RAB6A, KAT6B, SLC39A7, PSME3, STX3, MRAP2, LCOR, ZNF117, HINFP, RPL37, OPA3, ZNF562, SRCAP, TRPS1, FES, KCNMA1, SEC14L3, MYRIP, PTPN21, PTPN1, DAO, HELQ, CLN8, TNKS2, TOMM70A, FBLN7, MLLT3, SFT2D2, CD164, LY75, TARDBP, C2, WASF3, ANXA2, TBC1D32, HOXB3, EZH2, SEPT7, DDX3X, HACE1, CTSO, PACS2, POLR3E, ZNF107, API5, NAA15, POU5F1B, ERGIC2, FKBP9, ZNF746, DCTN2, ABI2, HDAC9, LPP, ATP2B1, NCL, SAFB, CHEK1, MYCBP2, PPP1R26, EPB41L1, HSPG2, SREBF2, DPF2, STK39, CDK8, FLII, SZT2, CHP1, SOGA1, DKC1, ZBTB37, IFIT2, TRPC7, GGA3, NXF1, SALL3, NAA50, PLCB1, CENPK, MAL2, LRRC59, STEAP1, ZNF676, SP4, SPINK5, ZC4H2, MAU2, NFAT5, NDUFAF4, THRAP3, GGA2, HNRNPAB, RSU1, CLCF1, PDCD10 , HPS1, HOMEZ, JUN, DCP2, SGSM1, PPARA, ACTR3, TM9SF1, BOD1, BPY2B, SHROOM4, TECPR1, MAOA, CDV3, CIAPIN1, TBC1D1, H2AFY, ACOT11, FMO6P, ATRIP, CCDC25, CBR1, ZNF493, MGMT, KRT77, SNAPC5, HOXA5, VEZT, NCKAP1L, WDR19, STRAP, STMN2, MTPN, FAN1, ZNF525, GUCY2C, CHD3, APLP2, BPY2, DENND4C, ZIC3, SHISA5, CSRNP3, RAB11FIP5, TIMP3, SMAD5, ZNF138, TRIB2, KRT6B, TIMM13, HYPK, AEN, BCAP29, SREK1, SATB1, KDM5A, UBR2 , KLK13, PHC2, PRKAR2A, ZNF708, UBE2N, ZNF592, ZNF599, CRB2, PNLDC1, PCCB , GLCE, MAPRE1, ZNF157, GPA33, FSHB, BCL2L2, NARF, CDC7, HSPA4L, LYPLA2, LSM11, DUSP3, MDM4, RNF146, VGLL2, ZNF646, ARID4B, NFE2L1, FXR2, PEF1, BROX, PLAG1, REV3L, EFNB1, USP14, BPY2C, CFH, GSTK1, ABCA5, TNIK, ZNF440, STX11, GRHPR, DTX1, KIF18B, PRICKLE4, CDK20, PRMT5 , STRC, CHD5, TPP1, ANXA11, CTCF, RAB22A, ZNF709, ZNF619, SAMD12, PRC1, TNR, WDR43, ZFHX4, ZRANB1, TCAIM, TGIF2, TXNDC17, ZNF483, OPA1, TGFBI, NCKAP1, GATAD1, HEBP2, ZNF202, SEC31B, JAGN1, CTLA4, UBE2B, UGT1A8, DNAJA2,ZNF674, CPEB3, SGK3, RPF2, TMEM214, SYT6, GLIPR2, CD84, GRWD1, PPP2R1B, DOK1, KMT2C, DGKI, IL17RD, CASP14, TRIM32, HMGB1, C1GALT1C1,*

		NGDN, ZNF766, ZBTB25, MRPL32, ATP8B4, TCN2, ITFG1, TOMM6, NR2F2, ILK, GNAI1, TMF1, PFKFB3, BSN, NEURL4, ZFAT, EFNA1, ITGB3
**Molecular Function**		
**GO category**	**p value**	**Target genes**
Ion binding (GO:0043167)	4.07×10^{-14}	*ZKSCAN5, HSPA13, JPH2, ARAP2, ESYT1, KSR2, ZNF423, GALNT7, CDH2, ARHGEF5, DIRAS1, CACNA2D3, ADAMTS4, PFAS, DSC2, TOP3A, PHF8, CLSTN3, USP49, ZNF616, RRAGD, ADAMTS19, DCTD, UBR1, ATXN7L3, LTA4H, FUS, SMAD2, ABCB8, TKT, ZNF76, WHSC1L1, ZNF468, MOK, ZNF83, DDX23, CALM1, ARF3, ITGA8, RORB, MORC4, CASK, ZNF688, CREB5, ATP1A2, ZNF131, ZDBF2, HHIP, ABCG4, SGPL1, CYP46A1, ADAT1, RAB6A, SPOCK1, KAT6B, MTMR4, STX3, ANGPT2, ZNF117, HINFP, RPL37, CA12, ZNF562, SRCAP, TRPS1, FES, KCNMA1, TNFRSF11A, MYRIP, PTPN1, DAO, ETNK2, PPP6C, ELTD1, EFHC1, AGAP1, HELQ, TNKS2, FBLN7, AASDH, C2, ANXA2, PGM3, RET, HEG1, MAP3K9, SEPT7, DDX3X, ZNF107, FKBP9, ZNF746, ACVR1, HDAC9, LPP, ATP2B1, PCDH17, CHEK1, MYCBP2, ATP5S, KIF21B, HSPG2, DPF2, STK39, CDK8, ACVR2B, CHP1, ZBTB37, SALL3, ITGA11, PIAS3, PLCB1, STEAP1, ZNF676, SP4, ZC4H2, SLC5A3, THRAP3, DCP2, N4BP2, PPARA, KIF26B, ACTR3, GPD2, TECPR1, QARS, MAOA, CIAPIN1, FMO6P, ZNF493, GLRA2, FAN1, ZNF525, GUCY2C, CHD3, APLP2, ZIC3, ACVRL1, TIMP3, SMAD5, ZNF138, TRIB2, TIMM13, KDM5A, UBR2, PHC2, PRKAR2A, ZNF708, UBE2N, ZNF592, ZNF599, CRB2, PCCB, ZNF157, TK2, RNF219, CDC7, HSPA4L, MTHFR, HUNK, FIGN, DDX55, DTNA, MDM4, RNF146, ZNF646, PEF1, CYP20A1, CBLL1, PLAG1, REV3L, THBS3, CFH, PRKAG2, GUCY1A2, ABCA5, TNIK, ZNF440, GRHPR, ST8SIA2, DTX1, KIF18B, PRICKLE4, CDK20, PDE5A, CHD5, TPP1, ANXA11, CTCF, RAB22A, ZNF709, ZNF619, MKRN3, ZFHX4, ZRANB1, TRIM44, ZNF483, BCL11B, OPA1, ABCC5, GATAD1, ZNF202, FRAS1, PRKG1, ITGA6, UBE2B, KMT2E, UGT1A8, DNAJA2, ZNF674, SGK3, SYT6, KMT2C, ADCY4, DGKI, TRIM32, ZNF766, ABCC6, ZBTB25, SLC46A1, ATP8B4, LACE1, TCN2, GLYCTK, NR2F2, ILK, GNAI1, PFKFB3, TNNI1, BSN, ZFAT, GRIN2B*
Enzyme regulator activity (GO:0030234)	0.01	*ARAP2, CDKN2C, PPP2R5E, WASL, CALM1, RALGAPA2, SPOCK1, PSME3, CSNK2B-LY6G5B—991, AGAP1, SERPINB7,ANXA2, TBC1D32, GRTP1, PPP1R26, CHP1, PLCB1, SPINK5, JUN, SGSM1, TBC1D1, H2AFY, NCKAP1L, ANKLE2, APLP2, TIMP3, TRIB2, PRKAR2A, CRB2, ARHGEF15, USP14, PRKAG2, TNIK, UGT1A8, MEGF10, TNFAIP8, DUSP19*
Nucleic acid binding transcription factor activity (GO:0001071)	0.01	*ZKSCAN5, ZNF616, PAX6, SMAD2, ZNF83, RORB, CREB5, ZNF131, ZNF117, HINFP, TRPS1, TARDBP, HOXB3, POU5F1B, ZNF746, TFDP2, NFAT5, HNRNPAB, JUN, PPARA, ZNF493, SNAPC5, HOXA5, NCKAP1L, ZNF525, ZIC3, CSRNP3, SMAD5, ZNF138, KDM5A, ZNF157, NFE2L1, PLAG1, CTCF, TGIF2, ZNF483, GATAD1, ZNF202, HMGB1, ZBTB25, NR2F2*
Enzyme binding (GO:0019899)	0.03	*CCNT2, UBXN7, CDKN2C, PTPN3, CDH2, ADAMTS4, PAX6, SMAD2, CALM1, HINFP, MYRIP, PTPN1, TNKS2, HACE1, ZNF746, ABI2, HDAC9, PPP1R26, CHP1, GGA3, PIAS3, PLCB1, GGA2, JUN, PPARA, H2AFY, DLG3, ANKLE2, ACVRL1, SMAD5, TRIB2, PRKAR2A, CRB2, MDM4, GCSAM, TSPAN17, PRIMA1, CASP10, PRKAG2, DTX1, PRC1, NCKAP1, UBE2B, KMT2E, UGT1A8, DUSP19, ILK, PPP1R3D, ITGB3*

Table S2. Downstream target genes, functional characteristics, and disease associations of shared regulatory transcription factors associated with hsa-miR-4715-3p and hsa-miR-5000-3p

Transcription Factor	Target Genes	Transcription Factor Functions	Associated Diseases
AR (Androgen receptor)	*ACTA1, AKR1C3, AKT1, ALDH1A3, ARG1, ARG2, ATAD2, BSG, BTG1, BTG2, CDK20, CDC6, CDH1, CDH11, CDH2, CDK6, CDKN1A, CLK3, CRH, CRISP1, CRISP3, CTNNB1, CYP7B1, EGFR, ELK4, EP300, ERBB2, ERBB3, ETV1, FKBP4, FN1, FOLH1, FOXA1, GNMT, GREB1, GTF2F1, HMMR, HMOX1, IFI16, IGF1R, IGFBP3, JUN, KISS1, KISS1R, KLF5, KLK1, KLK2, KLK3, KLK4, MED1, MID1, MUC1, MYC, NAT1, NDRG1, NFKB1, NKX3-1, NOV, NRAS, PC, PSCA, PEBP1, PIGR, PIP, PMEPA1, PNRC1, PTEN, PTGS2, PTPN1, RELA, REST, SEPT7, SERPINB5, SESN1, SRD5A3, STIM1, TBP, TCF7L2, TGFA, TH, TJP1, TMPRSS2, TRPS1, TSC1, TSC2, UCN, UGT2B15, USP26, VEGFA, VIM, WEE1, WNT7B, XIAP*	Regulates eukaryotic gene expression and plays an important role in controlling cellular proliferation and differentiation within target tissues.	Malignant neoplasm of prostate, Malignant neoplasm of breast, Carcinoma of the Large Intestine, Adenocarcinoma, Malignant tumor of colon, Malignant neoplasm of urinary bladder, Melanoma, Malignant neoplasm of stomach, Malignant neoplasm of ovary, Primary carcinoma of the liver cells, Embryoma, Esophageal Neoplasms, Malignant neoplasm of pancreas, Endometrial Carcinoma.
ARNT (Aryl hydrocarbon receptor nuclear translocator)	*AHR, BHLHE40, BRCA1, CA9, CCNE1, CDK2, CTSD, CYP1A1, CYP1B1, EGLN2, EPO, ESR1, FOS, GAD1, HIF1A, JUN, MFSD2A, RELB, UGT1A6, VEGFA*	Regulates the transcription of genes involved in angiogenesis, hematopoiesis, metabolism, cell motility, and immune modulation.	Breast cancer, Embryoma, Malignant neoplasm of ovary, Neoplasm Metastasis, Carcinoma of the Large Intestine, Malignant neoplasm of prostate, Endometrial Carcinoma, Breast Carcinoma, Malignant neoplasm of endometrium, Endometriosis, Renal Cell Carcinoma.
ATF3 (Activating transcription factor 3)	*ADIPOR1, ADIPOR2, AK4, ASNS, CCL5, CD82, DDIT3, GDF15, ID1, IFNG, PCLAF, LDLR, MMP2, PENK, SELE, TP53*	Regulates cellular stress responses by binding to CRE sequences and repressing transcription of target genes.	Diabetes Mellitus, Non-Insulin-Dependent Malignant neoplasm of prostate, Malignant neoplasm of stomach Cancer, Rheumatoid Arthritis, cardiovascular system disease, Malignant neoplasm of breast
CTCF (CCCTC-binding factor)	*ANG, APP, ASAP2, ATXN7, BBC3, BCL6, BRCA1, CCND1, CELSR3, CSF2, DMPK, ETNK2, H19, PBXIP1, IGF2, IPO13, IRAK2, MEG3, MYC, NANOG, NF1, PAX6, PTPRG, RAD21, RARRES1, RBL2, SIX5, SLC6A4, TERT, WT1*	Regulates gene expression and three-dimensional chromatin organization by mediating chromatin looping and establishing boundaries between regulatory regions.	Burkitt's tumor or lymphoma, cancer, Leukemia, Retinoblastoma, Myeloid leukemia, acute, Glioblastoma, Congenital Abnormality, Carcinoma, Transitional Cell, Adenovirus Infections, Ovarian, Dependence, Carcinoma, Carcinoma of the Large Intestine, Cervical Squamous Cell Carcinoma, Herpangina, Glioma.
E2F1 (E2F transcription factor 1)	*ABCB1, APAF1, AR, ASF1B, ATAD2, ATG5, ATM, AURKA, AURKB, BBC3, BCL2, BIRC5, BMI1, BRCA1, CASP7, CCL3, CCNB1, CCND1, CCNE1, CD2AP, CDAN1, CDC6, CDCA7, CDK1, CDKN1A, CDKN1C, CDKN2A, CHEK1, CHEK2, CREBBP, CTNNBIP1, CTSV, CUX1, DAPK2, DBF4, DDIT3, DHFR, DIRAS3, DNMT1, DOK1, DRAM1, DROSHA, DUSP1, E2F1, E2F6, ECT2, ERCC1, ERCC5, ERICD, ETV4, FANCA, FGFR1, FHIT, FLT1, FMN2, FOXM1, FSHR, FZD1, GINS3, H19, HIC1, HMGA1, HSPA5, IL1B, IL23A, IRF3, ISYNA1,*	Regulates cell-cycle progression, DNA replication, cell proliferation, and apoptosis by activating transcription of target genes.	Embryoma, Malignant neoplasm of breast, Leukemia, Malignant neoplasm of lung, Malignant neoplasm of stomach, Carcinoma of the Large Intestine, Malignant neoplasm of prostate, Melanoma, Stomach Carcinoma, Retinoblastoma, Primary carcinoma of the

	KDR, KIF2C, KIR3DL1, LRBA, MAP1LC3A, MAP3K5, MAPK8, MCL1, MCM5, MCM8, MCPH1, MDM2, MEFV, MIR202, MT1G, MUC4, MYB, MYBL2, MYC, MYCN, MYH10, NELL2, NOX4, NPAT, NRIP1, PCNA, PCSK6, PDK4, PEG10, PIN1, PLK1, PNRC2, PPARG, PPP1R13B, PTPRO, RACGAP1, RAD51, RASGEF1B, RASGRP1, RB1, RBM38, RNF2, RPA2, RRM1, RRM2, RRP1B, SERPINB5, SERPINE1, SIVA1, SKP2, TERT, THBS1, TLR3, TNF, TNFSF11, TOP2A, TP53, TP53BP2, TP53INP1, TP73, TYMS, UHRF1, ULK1, VEGFA, WWOX, XRCC1, ZNF350		liver cells, Malignant neoplasm of pancreas, Squamous cell carcinoma, Malignant neoplasm of ovary, Rheumatoid Arthritis, Malignant tumor of colon
E2F4 (E2F transcription factor 4)	*AR, AURKB, BIRC5, BRCA1, CCNB1, CCND1, CDK2, CUX1, DIRAS3, HCFC1, PCLAF, LIN54, LTBP4, MAP3K5, MCM10, MYC, PEG10, PLK1, PLK4, PPARG, RAD51, TOPBP1, TTK*	Regulates cell-cycle progression and DNA replication by controlling the transcription of target genes.	Embryoma, Breast Carcinoma, Renal Cell Carcinoma, Malignant neoplasm of breast, Leukemia Melanoma, Myeloid leukemia, acute Esophageal Neoplasms, Retinoblastoma.
ELF4 (E74 like ETS transcription factor 4)	*CXCL8, HBD, MDM2, MMP9, TP53*	Regulates gene transcription, immune cell function, inflammatory responses, cell proliferation, and senescence.	Colorectal Neoplasms, Liver Metastases, Hodgkin's disease, Malignant neoplasm of urinary bladder, Malignant neoplasm of cervix uteri, Metastasis to Lymph Nodes, Lymphoma, Leukemia, Lymphocytic, chronic Glioblastoma, Endometrial Carcinoma, Breast Carcinoma, Renal Cell Carcinoma, Malignant Glioma, Myeloid leukemia, Squamous cell carcinoma.
ERG (ETS transcription factor ERG)	*ADAMTS1, CDH5, CRISP3, CXCL8, CXCR4, ENG, EPB41L3, EPB41L4B, ERG, FGF2, HIST1H4L, HMOX1, HPGD, ICAM1, ICAM2, ILK, KCNN2, PIM1, SOX4, SPP1, TDRD1, TFF3, TMPRSS2, VIM, VWF, WNT11, ZEB1*	Regulates gene transcription and chromatin structure, contributing to cell proliferation, differentiation, angiogenesis, inflammation, and apoptosis.	Malignant neoplasm of prostate, Adenocarcinoma of Prostate, cancer, Hepatitis C, chronic, Myoma, Biliary Atresia, disease, Yersinia infections, Tuberculosis, Prostatic Neoplasms, Malignant neoplasm of breast, Malignant tumor of colon, Cerebral Infarction, Pancreatitis, relapsing, Pre-Eclampsia, Myelofibrosis, Melanoma, Malignant mesothelioma.
ESR1 (Estrogen receptor 1)	*ABCG2, AHR, AMH, AR, AVP, BCL2, BLM, BRCA1, BTG2, CCNA2, CCND1, CD24, CDH1, CDK4, CDKN1A, CDKN1B, CEBPB, CHAT, CRH, CRHBP, CTNNB1, CTSD, CXCL12, CYP19A1, CYP1A1, CYP1B1, CYP2C19, DCT, E2F1, EGFR, ESR1, ESRRA, F12, FLT1, FOS, FOXP1, GREB1, HSPB1, HTRA2, JAK2, JUN, JUNB, KDR, KRT19, MDM2, MICB, MMP13, MTA3, MYC, NQO1, NR5A2, NRF1, OXT, PELP1, PGR, PLAC1, PMAIP1, PTMA, RARA, RET, RUNX2, SERPINB9, SERPINE1, SP1, TAC3, TERT, TFF1, TGFA, TP53, TYMS, UGT1A4, UGT2B15, VEGFA, YWHAQ, ZEB1, ZFHX3*	Regulates estrogen-dependent gene transcription and signaling, influencing cellular proliferation and differentiation.	Malignant neoplasm of breast, Malignant neoplasm of prostate, Malignant neoplasm of ovary, Embryoma, Malignant neoplasm of stomach, disease, Primary carcinoma of the liver cells, Breast Carcinoma, Renal Cell Carcinoma, Carcinoma of the Large Intestine, Neuroblastoma, Stomach Carcinoma.
EZH2 (Enhancer of zeste 2 polycomb	*ADAMTS1, ADRB2, ALDH1A1, APC, AXL, BRCA1, CCND1, CCNE1, CDH1, CDKN1A, CDKN2A, CDKN2B, CIITA, CPEB1, DACT3, FBXO32, FOXC1, HDAC1, HOXB13, IGFBP1, KPNB1,*	Mediates epigenetic gene silencing through histone H3K27	Malignant neoplasm of breast, Carcinoma of the Large Intestine, Malignant neoplasm of prostate,

repressive complex 2 subunit)	*MMP2, MST1, MYC, PAX8, PCNA, PEBP1, PIP5K1C, POLR3D, PRL, PTEN, RPLP0, RUNX3, SATB1, SFRP1, SNAI2, SSTR1, TP53, TWIST1, VDR*	methylation, thereby regulating transcription, cell identity, and differentiation.	Embryoma, Squamous cell carcinoma, Primary carcinoma of the liver cells, Endometrial Carcinoma, Stomach Carcinoma, Carcinoma in situ, Epithelial ovarian cancer, Melanoma.
FLI1 (Fli-1 proto-oncogene, ETS transcription factor)	*CCK, COL1A2, CTGF, EP300, ERG, EYA3, FLI1, FOXM1, GATA1, GP6, GP9, HDAC1, HMOX1, ID2, IGFBP3, IL10, ITGA2B, MIR708, MMP1, MPL, PF4, PHLDA1, TERT, TGFBR2*	Acts as a sequence-specific transcriptional activator by binding to target DNA regions and regulating gene expression.	Ewings sarcoma, Malignant neoplasm of breast, Thrombocytopenia, Aplastic Anemia, Malignant neoplasm of prostate, Systemic Scleroderma, Malignant neoplasm of stomach, Melanoma, Fibromyalgia.
GATA2 (GATA binding protein 2)	*ADH1A, ANGPT2, AR, CYBB, DPYSL2, EPO, ERVW-1, FCER1A, GATA1, IL1RL1, MS4A2, NOS3, PECAM1, PPARG, PRG2, SPI1, ST2, TSHB, WT1*	Regulates gene transcription involved in the development, differentiation, and proliferation of hematopoietic and endocrine cells.	Myeloid leukemia, acute, Endometrial Carcinoma, Endometriosis, Unspecified myeloid leukemia, Yersinia infections, Thrombocytosis, Leukemia, Gastrointestinal Stromal Tumors, Retinoblastoma, Renal Cell Carcinoma, Fibroid Tumor, Malignant neoplasm of thyroid.
HIF1A (Hypoxia inducible factor 1 subunit alpha)	*ABCB1, ACE, ACE2, AGTR1, ALAS2, ALDOA, ALOX5AP, APEX1, AR, ARNT, ARNT2, ASS1, BAX, BID, BNIP3, CA9, CCND1, CCR7, CDH1, CTGF, CXCL12, CXCR4, E2F3, EDN1, EDNRB, EGLN1, EGLN3, ENO1, EPO, ERCC2, FAM162A, FGF2, GAPDH, GCK, HBEGF, HIF1A, ITGB2, KLK3, LDHA, LEP, LOX, MET, MICA, MIF, MMP2, MUC1, MUC3A, NR3C1, NR4A1, NT5E, PFKFB3, PFKFB4, PGK1, PIK3CA, PLAU, PPARA, PPP1R3C, PTGES, RECK, RORA, SDHB, SERPINE1, SETD2, SLC29A1, SLC2A1, SOCS1, STAT3, TERT, TFRC, TGFB3, TIMP2, TLR2, TLR6, TUBB3, TWIST1, TWIST2, VEGFA, VEGFB, VHL, VIM, WASF3, XPC, ZNF217*	Regulates cellular adaptation to hypoxia by activating genes involved in angiogenesis, glucose metabolism, and oxygen homeostasis.	Malignant neoplasm of breast, Renal Cell Carcinoma, Malignant neoplasm of stomach, Carcinoma of the Large Intestine, Squamous cell carcinoma, Primary carcinoma of the liver cells, Malignant neoplasm of prostate, Embryoma, Malignant neoplasm of urinary bladder, Malignant neoplasm of ovary, Malignant neoplasm of lung, Endometriosis, Atherosclerosis,.
JUN (Jun proto-oncogene, AP-1 transcription factor subunit)	*ABCB1, ACTA1, ALOX12, APOC3, APOM, APP, AR, ATF3, BATF3, BCL2L1, BECN1, BEX2, BRCA1, CA2, CCK, CCL2, CCL5, CCND1, CD82, CDK5R1, CDKN1A, CREM, CSF1, CSF2, CSTA, CTGF, CTSL, CXCL8, CYP11A1, CYP19A1, CYP1A2, CYP2J2, DCN, DDIT3, DDX21, EDN1, EGFR, ELN, ETS1, ETS2, EZH2, EZR, F3, FAS, FASLG, FGF7, VEGFD, FOSL1, GCLC, GJA1, GSTP1, HEY1, IBSP, IFNB1, IFNG, IL12A, IL12B, IL1A, IL1B, IL2, IL23A, IL24, IL3, IL5RA, IL6, ITGAX, ITGB8, JUN, KRT16, LBP, LDHA, LGALS3, LOR, MAP3K1, MAPK8, MAT2A, MEF2D, MELTF, MGMT, MGP, MMP1, MMP12, MMP13, MMP2, MMP20, MMP3, MMP7, MMP9, MSR1, MYB, MYC, NAMPT, NEFL, NEIL1, NFKB2, NGF, NOS2, NOS3, NOX5, NPY, NQO1, NTS, OPRM1, OXTR, PCK2, PDHA1, PDK1, PENK, PGR, PLAT, PLAU, PLAUR, POLD2, PPARA, PPARD, PTGS2, PTN, RARA, RARB, RARG, RELB, REN, RHOB, RUNX2, SLC19A1, SMAD7, SMN1, SOD2, SOX7, SPI1, SPRR1B, SPRR3, STAR, STMN1, SYK, TERT, TFF1, TIMP2, TNC, TNF, TNFRSF10A, TNFRSF1B, TP53, TP73, TXN, UGT2B15, VCAM1, VDR, VEGFA*	Regulates AP-1-dependent gene transcription and contributes to cell proliferation, differentiation, apoptosis, and stress responses.	Malignant neoplasm of prostate, Melanoma, Malignant neoplasm of breast, Embryoma, Carcinoma of the Large Intestine, Malignant neoplasm of stomach, Squamous cell carcinoma, Malignant neoplasm of pancreas, Rheumatoid Arthritis, Malignant neoplasm of ovary, Primary carcinoma of the liver cells, Endometriosis, Malignant neoplasm of urinary bladder, Malignant neoplasm of lung, Glioblastoma, Infection, Endometrial Carcinoma, Stomach Carcinoma, Degenerative polyarthritis, Renal Cell Carcinoma, Leukemia, Glioma, Diabetes

			Mellitus, Non-Insulin-Dependent.
KLF4 (KLF transcription factor 4)	*ALPI, ATF3, BDKRB2, BIRC5, CCNB1, CCND1, CD14, CDH1, CDH5, CDKN1A, CDKN1B, CDKN1C, CDX2, CYP1A1, GDF15, GPA33, HBA1, HBA2, HDC, HSPA8, IFITM3, IL1B, IL6, IVL, KRT19, LAMA1, LAMA3, LXN, MMP2, NANOG, ODC1, PFKP, RARA, SOD1, TAGLN, THBD, TP53, VDR, VEGFA, ZNF750*	Regulates gene transcription as both an activator and repressor, contributing to stem cell maintenance, cellular differentiation, and p53 regulation.	Carcinoma of the Large Intestine, Squamous cell carcinoma, Malignant neoplasm of stomach, cancer, Thyroid carcinoma, Malignant neoplasm of breast, Carcinoma of Nasopharynx, Stomach Carcinoma.
MAX (MYC associated factor X)	*BRD7, FASLG, MYC, ODC1, TERT*	Regulates gene transcription through interaction with MYC or MAD proteins, acting as either a transcriptional activator or repressor.	Carcinoma of the Large Intestine, Hepatitis C, Transitional Cell, Malignant neoplasm of urinary bladder, Stomach Carcinoma, Malignant neoplasm of cervix uteri, Multiple Myeloma, Carcinoma, Malignant neoplasm of pancreas, Malignant tumor of colon, Leukemia, Malignant neoplasm of breast
PGR (Progesterone receptor)	*ABCG2, ATP1B1, BCL2, CCND1, CYP19A1, DUSP1, E2F1, EGFR, ERBB2, ESR1, FOXP3, HLTF, IGFBP1, IL10, IRS2, KLK3, KLK4, MYC, PLD1, PTGS2, RELA, RLN1, RLN2, VEGFA, YWHAQ*	Regulates progesterone-dependent gene transcription and influences cellular proliferation and differentiation.	Malignant neoplasm of prostate, Malignant neoplasm of breast, Malignant neoplasm of ovary, Malignant neoplasm of stomach, Embryoma, Malignant tumor of colon, Uterine Fibroids, Melanoma.
POU2F1 (POU class 2 homeobox 1)	*AR, BDKRB1, CAPN6, CCND1, CDX2, GADD45A, GATA1, GCM1, GFI1B, GNRH1, HBG1, HIST2H2BE, HPGDS, ID1, IL2, IL2RA, IL4, IL5, LCT, LGALS4, MAD2L1, PLAU, POU2AF1, PTGIR, RPTOR, RRM2B, SDC4, SNAI2, SNAPC1, SPP1, ST6GAL1, TK1, TRA, TWIST1, TXNRD1, UGT2B7, VCAM1, VWF*	Regulates gene transcription by binding to octamer motifs and modulates transcriptional activity of steroid hormone receptors, including AR and PGR.	Malignant neoplasm of stomach, Embryoma, Malignant neoplasm of pancreas, Carcinoma of the Large Intestine, Malignant neoplasm of breast, Squamous cell carcinoma, disease, Malignant neoplasm of prostate.
RUNX1 (RUNX family transcription factor 1)	*ALOX12, BAALC, BPI, CCL3, CCND2, CD34, CD6, CSF1, CSF1R, CSF2, ELANE, FHL2, GP1BA, IL2, IL3, ITGA2B, ITGB2, JUN, KLF4, KLK3, LGALS3, MAD2L1, MECOM, MIR27A, MPL, MPO, MYH10, MYL9, NCAM1, NF1, NFKB1, PF4, PIK3CD, PRKCQ, RELA, SLC22A4, SPI1, SYMPK, TIMP1, VEGFA*	Regulates target gene transcription as part of the core-binding factor complex and contributes to hematopoietic development, immune cell differentiation, and T-cell function.	Myeloid leukemia, Malignant neoplasm of stomach, Malignant neoplasm of prostate, Malignant neoplasm of pancreas, Embryoma, Malignant neoplasm of breast, Carcinoma of the Large Intestine, Multiple Myeloma, Hodgkin's disease.
STAT1 (Signal transducer and activator of transcription 1)	*ACAT1, APP, BAX, CCL2, CCL3, CCR1, CD22, CD40, CD40LG, CD86, CDKN1A, CEBPE, CFTR, CHAT, CIITA, CTSB, CTSL, CXCL10, EDN1, EGFR, FAS, FCGR1A, FCGRT, FGF2, FGFR3, FOXP3, GAST, GLS, HLA-E, HMOX1, HSP90AA1, ICAM1, IFIT3, IFNA1, IFNB1, IFNG, IFNLR1, IL10, IL1B, IL1R1, IL27, IL2RA, IL6, IRF1, IRF7, IRF8, IVL, JAK2, KRT17, LCN2, LY96, MMP13, MMP9, MUC1, MUC4, MVP, NOS2, NOX1, NOX4, NOX5, NR1H4, OPRM1, PIM1, PLSCR1, PML, PPARA, PRKCE, PSMB9, PTGS2, S100A10, SCARB1, SMARCA4,*	Mediates interferon- and cytokine-dependent signaling and regulates transcription of genes involved in immune responses, inflammation, and antiviral defense.	Rheumatoid Arthritis, Malignant neoplasm of breast, Squamous cell carcinoma, Malignant neoplasm of prostate, Asthma, Degenerative polyarthritis, Melanoma, Myeloid leukemia, acute, Systemic lupus erythematosus, chronic, Carcinoma of the Large Intestine, Endometriosis,

	SOCS3, STAT2, STAT3, TAP1, TBX21, TIMP1, TLR3, TNFSF13B, TP53, UPP1, VIP, XAF1		Diabetes Mellitus, Primary carcinoma of the liver cells.
STAT3 (Signal transducer and activator of transcription 3)	*A2M, ABCA1, AKAP12, AKT1, AR, BCL2, BCL2L1, BCL6, BIRC5, CCL11, CCL2, CCND1, CCND2, CCND3, CD46, CDH1, CDK4, CDKN1A, CDKN1B, CFB, CFLAR, CHI3L1, CISH, COPS5, CRP, CSRP1, CTGF, CXCL8, CYP19A1, CYR61, DDIT3, DIRAS3, DNMT1, EGFR, EPAS1, ESR2, ETV6, F2R, FAAH, FAS, FGF1, FGF2, FGG, FGL1, FLT3, FOS, GAST, GFAP, GREB1, HAMP, HGF, HIF1A, HMOX1, HP, HSPA4, HSPB1, ICAM1, IFNAR1, IFNG, IKBKE, IL10, IL11, IL1RN, IL2, IL21, IL2RA, IL6, IRF1, JAK2, JUNB, KLF11, KRT17, LCAT, LEP, LGALS3BP, LTBP1, MCL1, MDC, MICA, MMP1, MMP10, MMP14, MMP2, MMP3, MMP7, MMP9, MUC1, MUC4, MUC5B, MYC, MYD88, NANOG, NLK, NME1, NOX1, NOX4, NOX5, NR2C2, OAS1, OPRM1, OSM, PAX3, PGF, PHB, PIAS3, PIM1, POU2F1, PPARA, PRDM1, PROS1, PSMB5, PTEN, PTGS2, PTPN13, PTPN6, ROR1, S100A11, S1PR1, SAA1, SALL4, SHH, SOCS1, SOCS3, STAT1, STK11, STS, TERT, TGFB1, TIMP1, TNFRSF10B, TP53, TP63, TRH, TWIST1, TWIST2, TYK2, UCP2, USP7, VDR, VEGFA, VEGFB, ZFP36*	Mediates cytokine- and growth factor-dependent signaling and regulates genes involved in cell proliferation, survival, inflammation, and immune responses.	Malignant neoplasm of prostate, Malignant neoplasm of breast, Primary carcinoma of the liver cells, Squamous cell carcinoma, Malignant neoplasm of stomach, Rheumatoid Arthritis, Endometriosis, Melanoma, Renal Cell Carcinoma, Multiple Myeloma, Embryoma, Malignant neoplasm of ovary, Carcinoma of the Large Intestine, Infection, Endometrial Carcinoma, Malignant tumor of colon, Malignant neoplasm of cervix uteri, Atherosclerosis.
STAT5B (Signal transducer and activator of transcription 5B)	*BCL2L1, BCL6, CCND1, CEL, ESR1, ESR2, IFNG, IGF1*	Mediates cytokine- and growth factor-dependent signaling and regulates genes involved in cell proliferation, survival, differentiation, and mammary gland development.	Malignant neoplasm of ovary, Malignant neoplasm of breast, Carcinoma of the Large Intestine, Uterine Fibroids, Endometriosis, Adrenocortical carcinoma, Malignant neoplasm of prostate, Late pregnancy, Embryoma, Primary Tumor, Malignant neoplasm of lung, Parkinson disease, Primary carcinoma of the liver cells, Lymphoma, Breast Cancer Stage IV.
TCF3 (Transcription factor 3)	*AICDA, BBC3, BTS1, CD4, CDH1, CDK6, CDKN1A, CR2, EXO1, IL5RA, INS, MYC, POU5F1, RAG1*	E-box-binding bHLH transcriptional regulator; regulates cell fate and differentiation and plays a role in neuronal and B-cell differentiation as well as mesenchymal-to-epithelial transition (MET).	Burkitt's tumor or lymphoma, Metaplastic Polyp, Cervical Squamous Cell Carcinoma, Carcinoma of the Large Intestine, Primary carcinoma of the liver cells, Kidney Failure, Laryngeal Squamous Cell Carcinoma, Retinoblastoma, Epithelial ovarian cancer, Lymphocytic, Malignant neoplasm of mouth, Embryoma, Carcinoma of Nasopharynx, Malignant neoplasm of breast.
TFAP2C (Transcription factor AP-2 gamma)	*CDKN1A, CRABP2, CSTA, ECM1, ERBB2, ESR1, GPX1, MMP2, RET, SULT1E1*	Regulates embryonic development, cell differentiation and chromatin-associated gene expression, and contributes to epigenetic regulation of ESR1 in breast cancer.	Metastasis to Lymph Nodes, Small cell carcinoma of lung, Embryoma, Medullary carcinoma of thyroid, Breast Carcinoma, Papillary thyroid carcinoma, Colorectal Neoplasms, Uterine Fibroids, Carcinoma of lung, Cervix carcinoma, Thyroid carcinoma, Malignant neoplasm of ovary.
